# Identification of a novel truncating *PALB2 *mutation and analysis of its contribution to early-onset breast cancer in French-Canadian women

**DOI:** 10.1186/bcr1828

**Published:** 2007-12-03

**Authors:** William D Foulkes, Parviz Ghadirian, Mohammed Reza Akbari, Nancy Hamel, Sylvie Giroux, Nelly Sabbaghian, Andrew Darnel, Robert Royer, Aletta Poll, Eve Fafard, André Robidoux, Ginette Martin, Tarek A Bismar, Marc Tischkowitz, Francois Rousseau, Steven A Narod

**Affiliations:** 1 Program in Cancer Genetics, Departments of Oncology and Human Genetics, McGill University, 546 Pine Ave West, Montréal, QC, Canada H2W 1S6; 2 Segal Cancer Centre, Sir M. B. Davis-Jewish General Hospital, 3755 Côte St-Catherine, Montréal, QC, Canada H3T 1E2; 3 The Research Institute, McGill University Health Centre, 1650 Cedar Avenue, Montréal, QC, Canada, H3G 1A4; 4 The CanGèneTest Research Consortium on Genetic Laboratory Services, Centre de recherche du CHUQ/HSFA, 10, rue de l'Espinay, Québec, QC, Canada, G1L 3L5; 5 Epidemiology Research Unit, Research Centre, Centre Hospitalier Université de Montréal (Hôtel-Dieu campus), 3850 St-Urbain, Montréal, Québec, Canada H2W 1T7; 6 Women's College Research Institute, University of Toronto, 790 Bay Street, 7^th ^Floor, Toronto, ON, Canada, M5G 1N8; 7 Centre de recherche du CHUQ – Hôpital St-François d'Assise, Centre Hospitalier Université de Laval, 10, rue de l'Espinay, Québec, QC, G1L 3L5; 8 Department of Surgery, Centre Hospitalier Université de Montréal (Hôtel-Dieu campus), 3850 St-Urbain, Montréal, Québec, Canada H2W 1T7; 9 Department of Pathology, McGill University, Duff Medical Building, 3775 University Street, Montreal, Quebec, Canada H3A 2B4

## Abstract

**Background:**

*PALB2 *has recently been identified as a breast cancer susceptibility gene. *PALB2 *mutations are rare causes of hereditary breast cancer but may be important in countries such as Finland where a founder mutation is present. We sought to estimate the contribution of *PALB2 *mutations to the burden of breast cancer in French Canadians from Quebec.

**Methods:**

We screened all coding exons of *PALB2 *in a sample of 50 French-Canadian women diagnosed with either early-onset breast cancer or familial breast cancer at a single Montreal hospital. The genetic variants identified in this sample were then studied in 356 additional women with breast cancer diagnosed before age 50 and in 6,448 newborn controls.

**Results:**

We identified a single protein-truncating mutation in *PALB2 *(c.2323 C>T, resulting in Q775X) in 1 of the 50 high-risk women. This variant was present in 2 of 356 breast cancer cases and in none of 6,440 newborn French-Canadian controls (*P *= 0.003). We also identified two novel new non-synonymous single nucleotide polymorphisms in exon 4 of PALB2 (c.5038 A>G [I76V] and c.5156 G>T [G115V]). G115V was found in 1 of 356 cases and in 15 of 6,442 controls (*P *= 0.6). The I76V variant was not identified in either the extended case series or the controls.

**Conclusion:**

We have identified a novel truncating mutation in *PALB2*. The mutation was found in approximately 0.5% of unselected French-Canadian women with early-onset breast cancer and appears to have a single origin. Although mutations are infrequent, *PALB2 *can be added to the list of breast cancer susceptibility genes for which founder mutations have been identified in the French-Canadian population.

## Introduction

Approximately 3% to 5% of breast cancer is estimated to be due to a dominantly inherited gene, and two breast cancer genes, *BRCA1 *and *BRCA2*, account for approximately 85% of families with four or more cases of breast and ovarian cancer [1]. Soon after these genes were discovered, it became apparent that specific founder mutations in these genes were present in different ethnic groups [2,3], including individuals of French-Canadian descent [4]. Five founder mutations account for 75% to 85% of all *BRCA1/2 *mutations in the French-Canadian population of Quebec [5,6]. However, for approximately one half of strongly hereditary breast cancer families, no *BRCA1/2 *mutation has been identified and other genes are believed to be important.

Many of the known hereditary breast cancer genes such as *BRCA1*, *BRCA2, CHEK2*, *ATM*, and *FANCJ/BRIP1 *work together in a DNA repair pathway [7], raising the possibility that other genes in this pathway also predispose to breast cancer. PALB2 ('partner and localizer of BRCA2') was recently identified to play an important role in facilitating the function of BRCA2; nearly 50% of BRCA2 is associated with PALB2 and more than 50% of PALB2 forms a complex with BRCA2 [8]. Association of BRCA2 with PALB2 appears to be essential for BRCA2 anchorage to nuclear structures and for its function in homologous recombination and double-strand break repair [8].

Studies including clinical samples from the UK [9,10], The Netherlands [11], Finland [12], and Canada [13] have confirmed that, like *BRCA2 *[14,15], *PALB2 *is both a breast cancer susceptibility gene and a Fanconi anemia gene. Disease-associated variants in *PALB2 *are rare in the UK population, with only approximately 1% of familial breast cancer being attributable to mutations in this gene, whereas 1% of all breast cancer in Finland is due to a single mutation (1592delT) in *PALB2 *[12].

We performed *PALB2 *mutation analysis in a series of unrelated French-Canadian women with breast cancer diagnosed at a single hospital in Montreal, Quebec, with the aim of identifying possible founder mutations and determining their contribution to breast cancer. We then estimated the population prevalence of each variant identified in a series of DNA samples from 6,448 newborns from Quebec City [16].

## Materials and methods

### Patients with breast cancer

All women were recruited from Hotel Dieu Hospital in Montreal and were seen in the breast clinic between October 2003 and May 2007. Women were selected for this study on the basis of (a) a diagnosis of first primary breast cancer under the age of 50 or (b) a diagnosis of breast cancer between the ages of 50 and 65 with at least one other case of breast or ovarian cancer in first- or second-degree relatives diagnosed at any age and (c) French-Canadian ancestry (all four grandparents). A total of 600 women were identified. Lymphocyte DNA extracted from the 600 French-Canadian women with breast cancer was undertaken using standard methods. All women had consented to participate in studies to identify potential breast cancer genes which had been subject to local ethical review. All women in the series had been previously tested for five French-Canadian founder mutations (*BRCA1*: 4666C>T and 2953delGTAinsC; *BRCA2*: 8765delAG, 6085C>T, and 3398delAAAG) [5]. Fifty women were selected for the pre-screen on the basis of their age and/or family history of breast and ovarian cancer. The median age of this group of women was 47.1 years (range 24 to 65 years). Three hundred fifty-six women were diagnosed with breast cancer under the age of 50 (median age 43 years, range 24 to 49 years). These two groups were analyzed for *PALB2 *mutations, as described below. The collection of cases was approved by appropriate institutional ethics review boards.

### Newborn controls

The population prevalence of each variant identified was estimated using a series of 6,448 newborn controls and a previously described and validated pooling strategy [16]. As reported previously [16], newborn cord blood leftovers were collected at the Hôpital St-François-d'Assise maternity unit (Quebec City) between 1996 and 2003 and were made anonymous and unlinked. DNA was purified from each sample and quantified, and pools of eight samples were prepared into bar-coded 96-well working plates using a QIAGEN BioRobot 3000 (Qiagen GmbH, Hilden, Germany). The final concentration of an individual genome in a pool was 7.5 ng/μL (on average), and 5 μL of each pool (or 38 ng of each genome) was used for genotyping. Genotyping was performed by allele-specific oligonucleotide polymerase chain reaction (PCR) in bar-coded 96-well microplates (Axygen Scientific, Inc., Union City, CA, USA) in a final volume of 25 μL containing 5 μL of DNA pools and 20 μL of PCR premix (1.5 μL 10× buffer [Qiagen GmbH], 200 μM of each deoxynucleoside triphosphate, 7.5 pmol of each primer, and 0.375 units of HotStarTaq DNA polymerase [Qiagen GmbH]) using a previously described quasi-homogeneous methodology [16] that is highly reproducible, robust, and affordable. Table 1 describes the primers and PCR conditions used for each mutation tested. Raw genotyping results were analyzed by in-house software in VC++ that determined genotypes and produced a result file with all pools. Individual samples from positive pools were tested separately using the same assay to confirm the presence of mutations. In all, 6,440 results were obtained for 6,448 samples for a call rate of greater than 99%. All positive pools were confirmed by individual sample analysis. The newborn study was approved by institutional ethics review boards in Quebec City and Montreal.

**Table 1:** Primers and conditions used for the newborn DNA population prevalence study

Mutation	Primers (5' to 3')	Wild-type allele conditions	Mutated allele conditions
Q775X	ACCAGTGGAGCCCTTTGAGTC-common primer GCCTGAACTGTCGAATTG-wild-type allele GCCTGAACTGTCGAATTA-mutated allele	Annealing temperature of 59°C for 35 cycles	Annealing temperature of 59°C for 36 cycles
I76V	TAGTCGCCCTGGTGAAATTAG-common primer CTCAGAACCTAAAAATAAAA-wild-type allele CTCAGAACCTAAAAATAAAG-mutated allele	Annealing temperature of 51°C for 36 cycles	Annealing temperature of 55°C for 38 cycles
G115V	TACTTGAGCCAAGGGGGAAAA-common CTGTTCTTTGTATAGGTAATC-wild-type allele CTGTTCTTTGTATAGGTAATA-mutated allele	Annealing temperature of 53°C for 35 cycles	Annealing temperature of 53°C for 39 cycles

### PALB2 sequencing

The *PALB2 *genomic sequence was obtained from the University of California at Santa Cruz Genome Browser, accession number NM_024675. Intronic primers were designed using Primer3 software (Whitehead Institute for Biomedical Research, Cambridge, MA, USA). Due to their large size, exons 4 and 5 were amplified in 4 and 2 amplicons, respectively; all primer sequences and annealing temperatures are listed in the online supplementary table from reference 13. We performed whole genome amplification of the 50 cases chosen for full sequencing (Repli-G mini kit; Qiagen GmbH) and confirmed all new variants identified by re-sequencing stock DNA in both directions. The PCRs were carried out in a volume of 50 μL, as previously described [13]. Sequence data were analyzed using the Lasergene SeqMan Pro sequence analysis software by DNASTAR, Inc. (Madison, WI, USA) and Chromas 2.31 from Technelysium Pty Ltd. (Helensvale, Australia).

### PALB2 exon deletion analysis

Multiplex ligation-dependent probe amplification (MLPA) was performed using the FANCD2-PALB2 kit (P057) from MRC-Holland b.v. (Amsterdam, The Netherlands) in accordance with the manufacturer's protocol. The MLPA assay covers the PALB2 exons, and in exon 1, one pair of probes covers 30 bases 5' of the ATG start site. The samples were run and analyzed on an ABI Prism 3100 (Applied Biosystems, Foster City, CA, USA) using fragment analysis tools.

### Testing for the new PALB2 variants in the under-50-years series

We developed a specific assay to look for the three novel variants we identified in the first phase of the study. The amplification refractory mutation system was employed for screening of the I76V and Q775X variants detected by direct sequencing in the first 50 French-Canadian cases with breast cancer. The inner and outer primer pairs used for genotyping of the I76V variant were 5'-TTTTCCTCCTCAGAACCTAAAAATACAA-3', 5'-TTTGATGTGTAACTTGTCATAAACACAGAC-3', 5'-CAGTGACCTTACTACTC ACAGCCTAAAA-3', and 5'-CAGTGACCTTACTACTCACAGCCTAAAA-3' at an annealing temperature of 52°C and for genotyping of the Q775X variant were 5'-ACTCAGTCTGTCTTGCCAGTGATACTACAC-3', 5'-GCTGGGCTGCCTGAACTGTCGAAGTA-3', 5'-TCTTAGGTACTACTCCAGC CTTTGGCCC-3', and 5'-TCCTGGCATGTGTTTCTACAGAGCTGAT-3' at an annealing temperature of 62°C. A restriction fragment length polymorphism assay was used for genotyping of the G115V variant. The primer pairs of 5'-TGCCTGAATGAAATGTCACTGATTCTT TC-3' and 5'-GGTGTCATCTGTTCTTTGTATAGGTcAT-3' were used for DNA amplification. Because there was no restriction enzyme cutting DNA at the site of mutation, the latter primer contained one mismatch nucleotide for producing a site identified by BtsCI enzyme. The length of the amplified DNA fragment was 296 base pairs (bp), and the enzyme cut the wild-type allele and produced two fragments of 273 and 23 bp whereas the mutated allele remained intact. All putative mutations were confirmed by sequencing.

### PALB2 haplotyping

For each of the three families that carry the Q775X mutation, we genotyped one individual for four microsatellite markers by PCR using an S^35^-dATP label. The markers used were D16S403, D16S481, and D16S3130 proximal to *PALB2*, and D16S417 distal to *PALB2*. (Primer sequences and PCR conditions are available in The Genome Database [17].)

### Pathology

The breast tumors from the three women carrying the identified truncating variant (Q775X) were re-analyzed by the study pathologist (TAB) and stained for estrogen receptor (ER), progesterone receptor (PR), and human epidermal growth factor receptor 2 (HER2-neu). Briefly, immunohistochemistry was performed using the NexES immunostainer (Ventana Medical Systems, Inc., Tuscon, AZ, USA). Formalin-fixed paraffin-embedded tissue of selected blocks from the three cases was stained by hematoxylin and eosin to confirm diagnosis. Primary antibodies for ER and PR (pre-diluted; Ventana Medical Systems, Inc.) (clone SP1 and IE2; rabbit monoclonal) and HER2 (Cell Signaling Technology, Inc., Danvers, MA, USA) (1:100) (clone 29D8; rabbit monoclonal) were used. Immunostaining was performed on 4-μm silane-coated slides (Sigma-Aldrich, St. Louis, MO, USA), which were dried overnight at 37°C and then dewaxed, rehydrated, and boiled (microwave) in EDTA (ethylenediaminetetraacetic acid) (pH 7.0) for antigen retrieval. Slides were incubated for 32 minutes at 37°C using the primary antibodies listed above. Diaminobenzidine was used as a chromogen, and slides were counterstained with hematoxylin before mounting. Negative controls were obtained by omitting the specific primary antibodies. Protein expression was assessed using a four-tiered system (0, negative; 1, weak; 2, moderate; and 3, high expression).

## Results

In the first phase of the study, we sequenced *PALB2 *in its entirety in 50 French-Canadian breast cancer patients selected on the basis of age of diagnosis or family history. Deletion analysis, using MLPA, was performed on 43 of the 50 samples. Sequence analysis identified one clearly pathogenic mutation, c.2323C>T (starting from the first ATG, or c.2523 C>T using GenBank nomenclature), resulting in protein truncation (Q775X). The proband carrying the Q775X mutation was diagnosed with invasive ductal breast cancer at age 54. We also identified two novel single nucleotide polymorphisms, c.5038 ATA>GTA (I76V) and c. 5156 GGA>GTA (G115V), both in exon 4 (Table 2). We also observed several single nucleotide polymorphisms, each of which has been previously identified in normal controls [10,13]. No deletions or duplications were identified by MLPA.

**Table 2:** *PALB2 *mutations identified in early-onset French-Canadian women with breast cancer and their frequencies in newborn controls

PALB2 variant	Frequency in cases (percentage)	Frequency in controls (percentage)	*P *value
Q775X	2/356 (0.56)	0/6,440 (0)	0.0027
I76V	0/356 (0)	0/6,442 (0)	-
G115V	1/356 (0.28)	15/6,442 (0.12)	0.99
All variants	3/356	15/6,442	0.065

In the second phase of the study, we analyzed 356 unrelated French-Canadian women diagnosed with invasive ductal breast cancer under age 50 for the three variants. We identified two carriers of the Q775X mutation (0.56%, 95% confidence interval [CI] 0.06% to 2.0%). The mutation was confirmed in both cases by re-sequencing of DNA from a new blood sample. This mutation is clearly pathogenic as it truncates the protein, and other truncating mutations occurring both before and after this amino acid are known to be capable of causing breast cancer. Notably, this mutation occurs before the WD-40 repeats, which are common protein-protein interacting motifs, and therefore, even if the Q775X generates a stable protein, the binding site for at least some of the PALB2 partners will have been lost. Nevertheless, the N-terminal putative coiled-coil domain remains intact, so it is possible in this scenario that some protein-protein interactions involving PALB2 will remain unaffected by this mutation.

We then genotyped four polymorphic microsatellite markers (D16S403, D16S481, and D16S3130 proximal to *PALB2*, and D16S417 distal to *PALB2*) and found that mutation carriers from the three families share a common allele at each locus. This is consistent with the possibility that Q775X is a founder mutation that arose on a common ancestral chromosome in the French-Canadian population, although further segregation data will be required to confirm this.

The two other novel variants, I76V and G115V, were studied in the entire series of cases. G115V was found in 1 of 356 early-onset cases, but I76V was not identified in any case. We then analyzed DNA from 6,448 newborns from Quebec City for the three variants identified in the first phase of the study. No carrier of the Q775X truncating variant was identified in these newborns, and the difference in frequencies between cases and controls reached statistical significance (*P *= 0.003). The other two *PALB2 *variants were not associated with breast cancer; G115V was identified in 15 of 6,442 newborns (odds ratio [OR] = 1.2; 95% CI, 0.02 to 7.9; *P *= 0.99), and I76V was not found in any control (Table 2).

**Figure 1:**
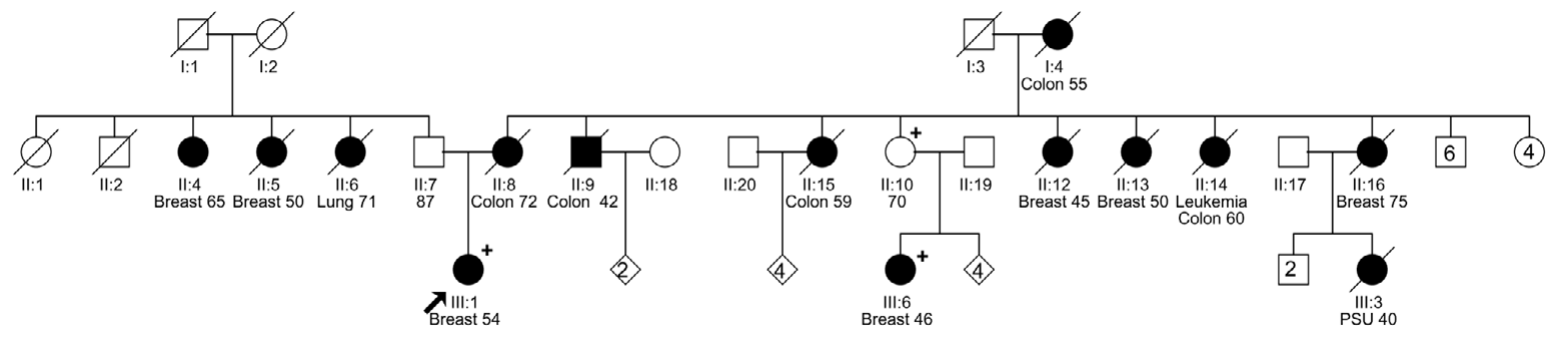
Pedigree of the P28031 family, whose index case carried a Q775X mutation. The proband was diagnosed with breast cancer at age 54. She has female relatives on both sides of the family who have been diagnosed with breast cancer (*n *= 2 on paternal side, *n *= 4 on maternal side), and the mutation was shown to segregate on the maternal side, with a 46-year-old affected cousin also found to be a carrier. Remarkably, there appear to be five relatives, including her mother, who have been diagnosed with colorectal cancer. Plus sign indicates Q775X mutation carrier. PSU, primary site unknown.

**Figure 2:**
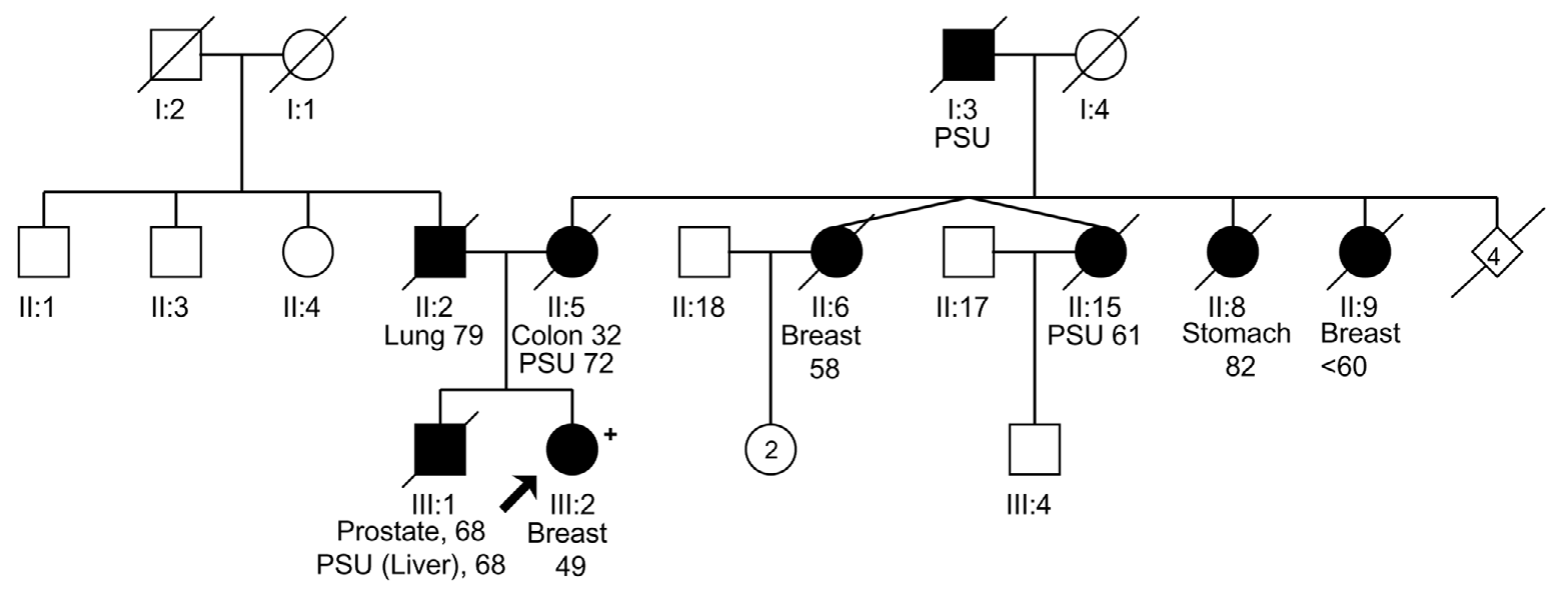
Pedigree of the P31030 family, whose index case carried a Q775X mutation. The proband was diagnosed with breast cancer at age 49. She reports two relatives diagnosed with breast cancer on her mother's side. She reports that her mother was diagnosed with colorectal cancer at age 32. Plus sign indicates Q775X mutation carrier. PSU, primary site unknown.

**Figure 3:**
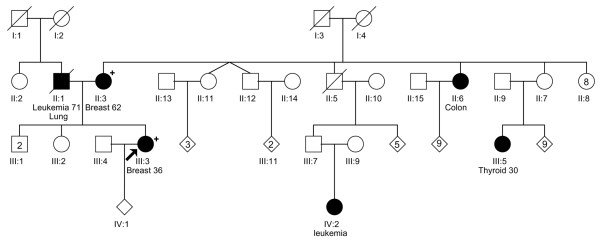
Pedigree of the P26007 family, whose index case carried a Q775X mutation. The proband was diagnosed with breast cancer at age 36. She does not report any breast tumors on her father's side of the family, but her mother (who was also found to carry the mutation) was diagnosed with breast cancer at age 62. There are also cases of leukemia, colon cancer, and thyroid cancer reported on this side of the family. Plus sign indicates Q775X mutation carrier.

The pedigrees of the three families in which the index case carried a Q775X mutation are shown in Figure 1 (Figure 1: P28031; Figure 2: P31030; and Figure 3: P26007). All women had a family history of breast cancer, and the BRCAPRO [18] scores for the probands were 0.03, 0.74, and 0.007, respectively. In two families, P28031 (Figure 1) and P26007 (Figure 3), it was possible to obtain samples from other affected family members and demonstrate segregation of the mutation. The tumors from the three women with truncating *PALB2 *mutations were studied in greater detail (Table 3). P28031 was diagnosed with a 1.5-cm grade 2 infiltrating ductal breast cancer at the age of 54. The lymph nodes were not involved by tumor. The tumor was ER^+^, PR^+^, and HER2-neu^-^. There was associated ductal carcinoma *in situ *(DCIS) (cribriform subtype, grade I/III). P31030 was initially diagnosed with DCIS (micropapillary and solid types, grade II/III) at age 49 in the left breast. The DCIS showed moderate expression of HER2 and was ER-moderate and PR-weak. The patient subsequently underwent a modified radical mastectomy for the right breast for a diagnosis of invasive ductal carcinoma grade 3 (maximum diameter 0.7 cm) with pagetoid spread and lymphovascular invasion. DCIS was also present (micropapillary and clinging types, grade III/III). Three out of 16 axillary lymph nodes showed metastatic carcinoma. The invasive tumor was ER^- ^and PR^-^. However, HER2-neu showed only weak expression. P26007 was diagnosed with an infiltrating ductal breast cancer at age 36. Measured clinically, the tumor was 3 cm. She was treated with neoadjuvant chemotherapy, and the post-treatment breast tissue showed no evidence of cancer in either the breast or in 14 lymph nodes. The pre-treatment biopsy showed invasive ductal carcinoma with medullary features, grade 3. Immunohistochemistry showed the tumor to be ER^-^, PR^-^, and HER2-neu^-^.

**Table 3:** Pathology findings in the breast specimens from three Q775X mutation carriers

Feature	P28031	P31010^a^		P26007
Histological type	IDC	Left breast DCIS only	Right breast IDC	IDC with medullary features

Size (cm) (invasive)	1.5	N/A	0.7	3.0^b^
BRE histological grade (IDC)	2	N/A	3	3
Associated DCIS	Cribriform, grade 1	Clinging micropapillary, grade 2	Micropapillary and clinging, grade 3	Not present
Nodal involvement	0/1 (sentinel)	N/A	3/16	0/14^c^
Estrogen receptor	+++ (>90%)	+ (20%)	-	-
Progesterone receptor	+++ (>90%)	++ (50%)	-	-
HER2-neu expression	-	++	- (+ in 10% CIS)	+ (10% of cells)

^a^Patient initially had excisional biopsy followed by modified radical mastectomy.

^b^Based on clinical measurement: the patient had neoadjuvant chemotherapy (doxorubicin and cyclophosphamide).

^c^Following neoadjuvant chemotherapy.

Key for immunohistochemical staining: +++, strongly positive; ++, moderately positive; +, weakly positive; -, negative.

BRE, Bloom-Richardson-Ellis; CIS, carcinoma *in situ*; DCIS, ductal carcinoma *in situ*; HER2-neu, human epidermal growth factor receptor 2; IDC, infiltrating ductal breast cancer; N/A, not applicable.

## Discussion

We have identified the *PALB2 *mutation Q775X to be a founder mutation for breast cancer in the French-Canadian population. To date, this mutation has been identified only in affected women of French-Canadian descent. The Q775X mutation appears to account for approximately 0.5% of breast cancer diagnosed in French-Canadian women under the age of 50. By contrast, five founder mutations in *BRCA1/2 *[5] account for approximately 4.3% of such women and *CHEK2*:1100delC is found in approximately 0.7% of these cases (P Ghadirian, A Robidoux, R Royer, P Zhang, S Zhang, E Fafard, M Costa, F Rousseau, G Martin, C Potvin *et al*., manuscript submitted; S Zhang, CM Phelan, P Zhang, F Rousseau, P Ghadirian, A Robidoux, W Foulkes, N Hamel, D McReady, M Trudeau *et al*., manuscript submitted). The novel *PALB2 *exon 4 missense variants I76V and G115V were both identified in the initial screening series, but I76V was not found subsequently. Although I76V seems to be rare, it is a relatively conservative change (Grantham score = 29; SIFT [sorting intolerant from tolerant] prediction = tolerated). G115V was found in both cases and controls, but the similar frequency and bioinformatic analyses (data not shown) indicate that this variant is highly unlikely to be associated with an increased risk for breast cancer. Moreover, exon 4 sequences are not essential for DNA repair associated with PALB2 function.

One of the strengths of this study is that the 356 cases studied in the second phase were not selected for family history. In addition, we were able to analyze a very large population-based sample of 6,448 newborns as a control group and thus can be sure that Q775X is not present at an appreciable frequency in the population (upper limit of 95% CI = 0.057%, or 1/1,745), using the Sign test with exact 95% CI.

PALB2 is a protein of approximately 130 kDa which interacts with BRCA2 and is a major determinant of the nuclear localization of BRCA2 [8]. Notably, it has recently been found to be a breast cancer susceptibility gene [10,12] and is a cause of Fanconi anemia, subtype N [9,11]. Until now, eight distinct truncating mutations have been associated with an increased risk for breast cancer (I76fs, L531fs, G796X, T841fs, A995fs, W1038X, N1039fs, and Y1183X) [9,10,12,13]. L531fs (c.1592delT) is a founder mutation in Finland but so far has not been seen outside of Finland. W1038X has been found in two separate families, and both N1039fs and Y1183X have been seen three times. These eight families were all ascertained in the UK but other details of their geographical origins have not been provided. The possibility of a link between these mutations and a defined subpopulation of the UK has not been excluded.

Previously, we [13] and others [12] showed that most breast tumors arising in *PALB2 *mutation carriers are ER^+^, PR^+^, and HER2^-^, like both *BRCA2*-related and sporadic breast cancer. The findings in the three *PALB2 *mutation carriers reported here (Table 3) suggest that these prior observations may not be universal; two of the three invasive tumors were ER^- ^and one of these was HER2^+^. Interestingly, one tumor had a medullary phenotype (on a pre-treatment biopsy), which is much more commonly a feature of *BRCA1*- than *BRCA2*-related breast cancer, although both tend to feature a continuous pushing margin [19].

This is the fifth study of the role of PALB2 in breast cancer; the findings are summarized in Table 4. Overall, it appears that *PALB2 *mutations are responsible for 1% to 2% of strongly familial breast cancer, but in Finland (and possibly in other founder populations), *PALB2 *mutations may account for up to 1% of all breast cancer. Deleterious *PALB2 *alleles are extremely rare in the general population, and only in Finland have any variants been found in individuals outside the setting of familial breast cancer. The OR for breast cancer in association with a truncating *PALB2 *mutation cannot easily be determined as the numbers of mutation carriers in each study are small, but the two studies [9,12] in which it has been calculated (using very different methods) found ORs of 2.3 and 3.9, respectively. A recent study from our group reported on one Quebec family of Scottish descent in which seven breast tumors occurred in three women, perhaps suggesting an increased penetrance [13]. Here, two of the three families were moderately suggestive of hereditary breast cancer and it appears that *PALB2 *generally behaves as a low- to moderate-penetrance breast cancer susceptibility gene. In this study, one of the three families reported five cases of colorectal cancer. In Figure 1, it can be seen that the mutation is present on the side of the family with colorectal cancer. All three families have at least one reported case. Notably, there are no reported cases of ovarian cancer. The possibility that *PALB2 *predisposes to colorectal cancer is worthy of further investigation.

**Table 4:** *PALB2 *mutation studies in breast cancer

Cases	Carriers/total probands studied	Controls	Carriers/total probands studied	Odds ratio (95% confidence interval; *P *value)
Unselected breast cancer (Finland)^a ^[12]	18/1,918 (0.9%)	Finnish Red Cross blood donors^b^	6/2,501 (0.2%)	3.91 (1.5–12.1; 0.002)^c^
Familial breast cancer^d ^(Finland) [12]	3/113 (2.7%)	-	6/2,501 (0.2%)	11.1 (1.8–52; 0.006)^c^
Familial breast cancer^e ^(UK) [10]	10/923 (1.1%) Female breast cancer only 9/908 (1.0%)	1958 Birth Cohort collection, all aged 48	0/1,084 (0%)	2.3 (1.4–3.9; 0.0025) <50 years: 3.0 (1.4–5.5, NS) >50 years: 1.9 (0.8–3.7, 0.35)
Strongly familial breast cancer (Canada) [13]	1/68^f ^(1.5%)	-	-	-
Breast cancer diagnosed in French-Canadian women <50 years of age^g ^(Quebec) (the present study)	2/356 (0.56%)	Newborns, Quebec City, 1996–2003	0/6,440 (0%)	-(3.4–∞, 0.0027)

^a^Mean age 57.7 years (range 23 to 95 years).

^b^Mean age 42.0 years (range 18 to 65 years).

^c^Recalculated from the original data.

^d^Sequenced and negative for BRCA1, BRCA2, CHEK2, and TP53. Sixty-five were strongly familial, and 48 were moderately strong, with at least 2 cases of breast and/or ovarian cancer in first- or second-degree relatives.

^e^Screening started with 96 BRCA1/2^- ^families. Thirty-five other genes have been screened in this series. The familial case sequenced was the affected proband (male or female) from a family with at least 3 cases of breast cancer diagnosed at any age. All probands were sequenced for *BRCA1/2 *and were also negative for large deletions. 'Non-UK ethnic groups' were excluded. The median age of the probands was 49 years.

^f^Carrier individual was of Scottish descent. All cases were sequence-negative for *BRCA1/2*.

^g^Median age 43 years (range 23 to 49 years).

NS: Not stated.

It is difficult to estimate the penetrance for genes in the class of *PALB2*. This is because the allele frequencies of the variants of the controls are too low to generate a robust estimate of the relative risk. (In more than 6,000 controls in our study, the Q775X allele was not found.) One might better estimate the penetrance by observing the families of cases identified to carry a *PALB2 *mutation, identified through the study of unselected cases. However (given the mutation prevalence of 0.5% in early-onset cases in this study), to generate a pedigree sample of sufficient size for analysis, it would be necessary to genotype many thousands of cases. In the French Canadian population of Quebec, founder mutations have been identified in *BRCA1*, *BRCA2*, and now *PALB2*. However, the full clinical relevance of *PALB2 *mutations is as yet uncertain. For this reason, genetic counseling solely on the basis of *PALB2 *mutation status will pose significant challenges, and widespread clinical testing for *PALB2 *mutations is unlikely to enter the clinical arena at the current time.

## Conclusion

We have identified a novel truncating mutation in *PALB2 *which appears to be a founder mutation in French Canadians. The mutation was found in approximately 0.5% of unselected French-Canadian women with early-onset breast cancer. No mutation carriers were identified in DNA from more than 6,000 newborn controls. *PALB2 *can be added to the list of breast cancer susceptibility genes for which founder mutations have been identified in the French Canadian population.

## Abbreviations

bp = base pairs; CI = confidence interval; DCIS = ductal carcinoma *in situ*; ER = estrogen receptor; HER2-neu = human epidermal growth factor receptor 2; MLPA = multiplex ligation-dependent probe amplification; OR = odds ratio; PALB2 = partner and localizer of BRCA2; PCR = polymerase chain reaction; PR = progesterone receptor.

## Competing interests

The authors declare that they have no competing interests.

## Authors' contributions

WDF planned and coordinated the study and wrote the manuscript. PG planned and organized the recruitment of all cases. MRA, NH, SG, NS, and RR conducted all genotyping. AD and TAB reviewed the pathology and performed the immunohistochemistry. EF recruited cases and provided clinical information. AP assisted with pedigree analysis. AR and GM referred cases and provided clinical information. MT assisted in planning and coordinating the study and revised the manuscript. FR designed and carried out the newborn screening study. SAN planned and coordinated the study and helped draft the manuscript. All authors read and approved the final manuscript.
